# Statistical limitations in ion imaging

**DOI:** 10.1088/1361-6560/abee57

**Published:** 2021-05-10

**Authors:** Charles-Antoine Collins-Fekete, Nikolaos Dikaios, Esther Bär, Philip M Evans

**Affiliations:** 1 Department of Medical Physics and Biomedical Engineering, University College London, Gower Street, London, United Kingdom; 2 Chemical, Medical and Environmental Science, National Physical Laboratory, Hampton Road, Teddington, United Kingdom; 3 Centre for Vision Speech and Signal Processing, University of Surrey, Guildford, United Kingdom

**Keywords:** ion tomography, particle imaging, range uncertainty

## Abstract

In this study, we investigated the capacity of various ion beams available for radiotherapy to produce high quality relative stopping power map acquired from energy-loss measurements. The image quality metrics chosen to compare the different ions were signal-to-noise ratio (SNR) as a function of dose and spatial resolution. Geant4 Monte Carlo simulations were performed for: hydrogen, helium, lithium, boron and carbon ion beams crossing a 20 cm diameter water phantom to determine SNR and spatial resolution. It has been found that protons possess a significantly larger SNR when compared with other ions at a fixed range (up to 36% higher than helium) due to the proton nuclear stability and low dose per primary. However, it also yields the lowest spatial resolution against all other ions, with a resolution lowered by a factor 4 compared to that of carbon imaging, for a beam with the same initial range. When comparing for a fixed spatial resolution of 10 lp cm^−1^, carbon ions produce the highest image quality metrics with proton ions producing the lowest. In conclusion, it has been found that no ion can maximize all image quality metrics simultaneously and that a choice must be made between spatial resolution, SNR, and dose.

## 1. Introduction

Energy-loss ion tomography is a relatively novel field of research that originated from (1) the necessity of predicting accurate relative stopping power (RSP) for treatment planning in hadron therapy (Paganetti 2012) and (2) the limitations imposed when trying to predict RSP with proton ions.

Indeed, proton imaging seems to be well suited to measure the stopping power of materials as it can directly measure the protons’ energy loss caused by crossing the material. This direct measurement is a considerable advantage when compared to other techniques that map various tissue properties (e.g. mass attenuation coefficient) to RSP through often empirical relationships. Furthermore, proton imaging demonstrates an advantageous noise to dose relationship (Schulte *et al* 2005, Depauw and Seco 2011), requiring less dose to achieve a suitable noise level than x-ray imaging. However, proton particles suffer from a series of Coulomb deflections as they cross the medium, inducing blurring in the reconstructed image (average simulated spatial resolution of 5 lp cm^−1^ compared to the accepted clinical x-ray CT standard of 10–11 lp cm^−1^ Collins-Fekete *et al* 2015).

Whereas proton imaging suffers from Coulomb scattering, due to the lower charge to mass ratio of protons, heavier ions scatter less through a medium and should therefore produce sharper images. Heavier ion imaging physics seems a viable choice to keep the RSP accuracy and signal-to-noise (SNR)/dose ratio promised by proton imaging while increasing the spatial resolution. Indeed, recent studies have proposed that helium imaging would be optimal (Collins-Fekete *et al* 2017, Gehrke *et al* 2018) to provide the highest spatial resolution/SNR balance amongst other ions, due to the helium nucleus relative stability, low charge to mass ratio, and abundance.

Recently, we performed an investigation of the statistical limitations of proton imaging, extending on preliminary work by Rädler *et al* (2018) and Schulte *et al* (2005), to propose a framework for predicting proton imaging SNR and spatial resolution against delivered dose and entrance energy for the different existing proton interactions (Collins-Fekete *et al* 2020). The methodology developed in that manuscript provided the means for comparing protons with heavier ions for imaging.

In this work, we extend the study of statistical limitations to the scenario of heavy-ion imaging, to investigate which ion beam choice maximises tomographic image quality metrics. To do so, we will investigate commonly available ions for imaging in terms of spatial resolution and the SNR/dose ratio for particles crossing a water cylinder where the beam energy has been chosen to produce either a fixed range in water or a fixed spatial resolution for each ion species.

## 2. Theory and model

The purpose of this work is to model a computed tomography scan using a set of projections from an ion beam which is passed through an object and to understand the relationship between the delivered dose, the noise, the signal and the spatial resolution for a set of ion species. For the purpose of image formation, we consider electromagnetic energy loss and noise, where the latter is separated into scattering noise and straggling noise. In term of simulation geometry, we will consider a fixed origin system where the beam travels along the *X*-axis through the phantom, and scatters along the orthogonal plane. However, since the scattering distribution in orthogonal planes is uncorrelated, we will consider only a beam of particle travelling along the *X* direction and scattering in the *Y*−*X* plane and ignore effects on the perpendicular plane. We will quickly summarize the model developed in our previous manuscript which forms the basis of this study (Collins-Fekete *et al* 2020). To do so, we will list the elements that contributes to the model and refer the reader to the previous study for further details. Briefly, the inverse problem in list-mode particle imaging is:

\begin{eqnarray*}\displaystyle \frac{{dE}}{{dl}}={\mathrm{RSP}}({\bf{r}}){S}_{w}(E({\bf{r}}))\leftrightarrow {\int }_{{\mathrm{\Gamma }}}{\mathrm{RSP}}({\bf{r}}){dl}={\int }^{{\mathrm{\Delta }}E}\displaystyle \frac{{dE}}{{S}_{w}(E({\bf{r}}))}={\mathrm{WET}},\end{eqnarray*}

where the suffix *Γ*represents the non-linear path taken by a particle through a medium, *S*_*w*_ represents the stopping power of a particle in water, Δ*E* is the energy loss, RSP is the RSP and WET is the water equivalent thickness. However, due to the stochastic nature of scattering, the path taken is uncertain and represented by a distribution of possible paths. That distribution is known as the beam scattering distribution and is a function of (1) the types of detectors and their configurations (Krah *et al* 2018) and (2) the particle charge/mass ratio and its velocity (Collins-Fekete *et al* 2017). Due to the probabilistic nature of this distribution, the energy loss also can be represented as the expected energy loss by particles following the multiple possible paths in the scattering distribution (Collins-Fekete *et al* 2020):

\begin{eqnarray*}{E}[{\mathrm{\Delta }}{E}]=\int {\mathrm{\Delta }}{E}({\mathrm{\Gamma }}(p,{Y}_{0},{Y}_{2})){\mathrm{dp}}.\end{eqnarray*}

In this equation, *Γ*represents a path within the beam scattering distribution for a given probability *p* as defined by Collins-Fekete *et al* (2020) and *Y*_0_ and *Y*_2_ are the entry and exit measurement vectors at fixed positions *x*_0_ and *x*_1_ (e.g. ${{Y}}_{0}=[{{y}}_{0},{\theta }_{{{y}}_{0}}]$ with ${\theta }_{{y}_{0}}$ the directional cosine). The WET is the quantity used to backproject RSP and is related to the expected energy loss as:

\begin{eqnarray*}{E}[{\mathrm{WET}}]={\int }_{{{E}}_{0}}^{{{E}}_{0}-{E}[{\mathrm{\Delta }}{E}]}\displaystyle \frac{{dE}}{{S}_{w}({E})}.\end{eqnarray*}

The reconstructed RSP can then be calculated as a back-projection of that quantity over the most likely path *Γ*convolved with a suitable filtering function as seen in Rit *et al* (2013).

### 2.1. Scattering and straggling noise

**Figure 1.**
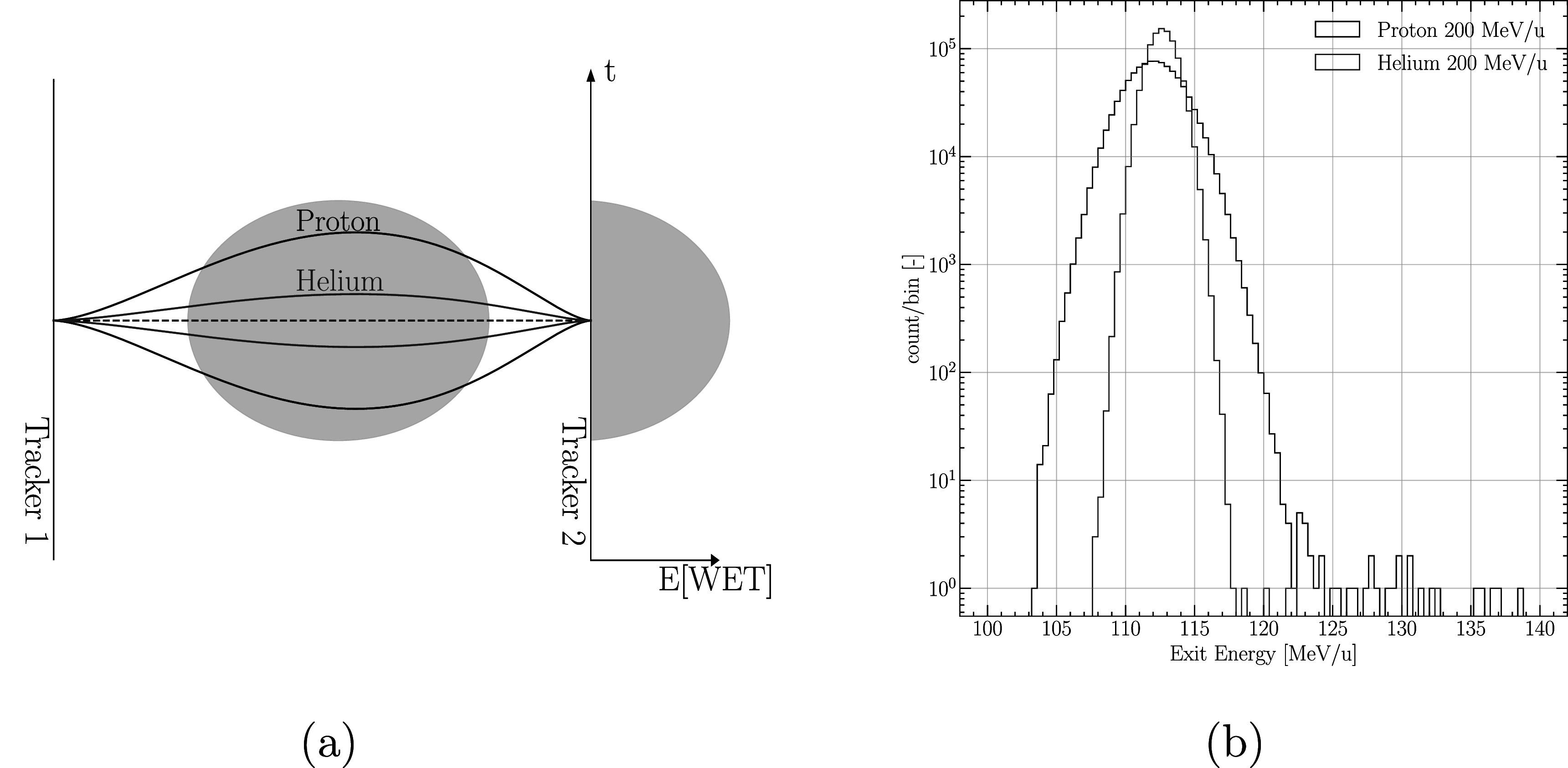
Representation of the various electromagnetic noise components involved in ion imaging. with example for protons and helium. (a) The black envelope describes the multiple Coulomb scattering (MCS) distribution for a beam of protons originating and ending at fixed points, crossing a cylindrical phantom, whereas the red envelope describes the same for a beam of helium particles. The MCS noise comes from the various paths and energy loss variations that ions can take within the distribution. (b) Probability distribution of residual energy for a 200 MeV/*u* proton (black) and helium (red) beam crossing a fixed 20 cm slab of water. The straggling noise originates from the statistical fluctuations in number of interactions and energy loss per interaction.

The scattering noise is demonstrated schematically in figure 1(a), and straggling noise is illustrated (from Monte Carlo simulations) in figure 1(b). The scattering distribution, which is equivalent to the probability distribution *p*, is assumed to be normal and represented by a two-dimensional Gaussian in space for a given plane. The Gaussian scattering distribution varies with depth and is represented in figure 1(a). For the plane *Y*−*X*, the dimensions of the Gaussian are position and direction respectively, e.g. *y* and *θ*_*y*_. The bivariate Gaussian co-variance matrix moments are given by:

\begin{eqnarray*}{\sigma }_{{\mathrm{scatt}}}^{2}{\left(x,{Z}_{i}\right)}_{n}={Z}_{i}^{2}{E}_{0}^{2}{\left(1+\displaystyle \frac{1}{9}{ln}\left({\int }_{{x}_{0}}^{x}\displaystyle \frac{{dx}^{\prime} }{{X}_{0}(x^{\prime} )}\right)\right)}^{2}{\int }_{{x}_{0}}^{x}\displaystyle \frac{{\left(x^{\prime} -{x}_{0}\right)}^{n}{dx}^{\prime} }{{{pv}}_{i}{\left(x^{\prime} \right)}^{2}{X}_{0}(x^{\prime} )},\end{eqnarray*}

where the term *X*_0_(*x*) represents the radiation length of the material at depth *x*, the empirical constant *E*_0_ =13.6 [MeV/*c*], and *Z*_*i*_ represents the atomic charge of the ion with the subscript *i* used to differentiate the ion species. The subscript *n* represents the different moments of the bi-variate Gaussian matrix, with *n* = 0 being the direction variance, *n* = 1 being the position-direction co-variance, and *n* = 2 the position variance. The term ${{pv}}_{i}(u^{\prime} )$ represents the momentum and velocity as a function of the depth in the material for the ion beam being investigated. The scattering noise in energy loss is defined as:

\begin{eqnarray*}{\sigma }_{{E}_{{\mathrm{out}}},{\mathrm{MCS}}}^{2}={\mathrm{E}}[{\mathrm{\Delta }}{E}^{2}]-{\mathrm{E}}{\left[{\mathrm{\Delta }}E\right]}^{2},\end{eqnarray*}

where the expectation operator is defined in equation (2). The second type of variance is the energy straggling, which represents the fluctuations in (1) the number of ion-electron collisions and (2) the energy loss in each collision, which is described as Tschalär (1968), Tschalär and Maccabee (1970):

\begin{eqnarray*}{\sigma }_{E,{\mathrm{strag}}}^{2}({E}_{{\mathrm{out}}})={k}_{1}^{2}({E}_{{\mathrm{out}}}){\int }_{{E}_{0}}^{{E}_{{\mathrm{out}}}}\displaystyle \frac{{k}_{2}(E)}{{k}_{1}^{3}(E)}{dE},\end{eqnarray*}

where *k*_1_ and *k*_2_ are defined as:

\begin{eqnarray*}{k}_{1}=\displaystyle \frac{{Z}_{i}^{2}K}{{\beta }^{2}(E)}\left[\mathrm{ln}\left(\displaystyle \frac{2{m}_{e}{c}^{2}}{I}\displaystyle \frac{{\beta }^{2}(E)}{1-{\beta }^{2}(E)}\right)-{\beta }^{2}(E)\right],\quad {k}_{2}={Z}_{i}{\eta }_{e}K\displaystyle \frac{1-1/2{\beta }^{2}(E)}{1-{\beta }^{2}(E)},\end{eqnarray*}

where *c* is the speed of light, *β* is the proton velocity relative to the speed of light, *η*_*e*_ is the relative electron density of the medium to the electron density of water, *m*_*e*_ is the relativistic electron rest mass, *I* is mean excitation energy of the medium, and the constant *K* = 170 MeV cm^−1^ combines various fixed physical parameters and the other parameters are defined as before. Both errors are assumed to be independent and the total error is the sum in quadrature of the individual standard deviation (${\sigma }_{{E}_{{\mathrm{out}}}}^{2}={\sigma }_{E,{\mathrm{strag}}}^{2}+{\sigma }_{E,{\mathrm{MCS}}}^{2}$). The noise of the WET backprojected quantity is found by propagating the errors through the energy-WET equation (equation (1)) such that:

\begin{eqnarray*}{\sigma }_{{\mathrm{WET}}}^{2}={\sigma }_{{E}_{{\mathrm{out}}}}^{2}{\left({\left.\displaystyle \frac{\partial {\mathrm{WET}}}{\partial E}\right|}_{E={E}_{{\mathrm{out}}}}\right)}^{2}=\displaystyle \frac{{\sigma }_{{E}_{{\mathrm{out}}}}^{2}}{{S}_{w}{\left({E}_{{\mathrm{out}}}\right)}^{2}}.\end{eqnarray*}

### 2.2. Signal to noise ratio against dose in the centre of a cylindrical object

The SNR equation in the centre of a cylindrical water phantom in ion tomography is identical to that which was found in Collins-Fekete *et al* (2020):

\begin{eqnarray*}\mathrm{SNR}={\left(\displaystyle \frac{3{{Ma}}^{2}}{{\pi }^{2}}\right)}^{1/2}\displaystyle \frac{{S}_{w}({E}_{{\mathrm{out}}})\sqrt{{N}_{D}}\hat{{\mathrm{RSP}}}}{{\sigma }_{{E}_{{\mathrm{out}}}}},\end{eqnarray*}

where SNR is the signal to noise ratio, *M* is the number of projections, *a* is the pixel size, *N*_*D*_ is the number of particles measured at the exit detector, $\hat{{\mathrm{RSP}}}$ is the reconstructed RSP and ${\sigma }_{{E}_{{\mathrm{out}}}}$ is the energy loss variation. The dose in a voxel of volume *a* ^3^ in the middle of a uniform water cylinder of diameter *d* can then be expressed as a function of the SNR:

\begin{eqnarray*}{D}_{c}=\displaystyle \frac{{\pi }^{2}{{\mathrm{SNR}}}^{2}{\sigma }_{{\mathrm{WET}}}^{2}{g}_{{ph}}^{{MC}}{g}_{{dt}}^{{MC}}{S}_{{MC}}({E}_{c},d/2)}{3{a}^{4}\rho {\hat{{\mathrm{RSP}}}}^{2}},\end{eqnarray*}

where *a* represents the pixel size, *D*_*c*_ represents the dose in the centre of the object, SNR is the SNR, ${\sigma }_{{\mathrm{WET}}}^{2}$ is the WET variance at the detector, *g*_*MC*_ represents the primary attenuation due to nuclear interaction between the middle voxel and the detection point, separated into phantom attenuation (*g* ^*MC*^_*ph*_) and detector attenuation (*g* ^*MC*^_*dt*_) components with ${g}^{{MC}}={g}_{{ph}}^{{MC}}\cdot {g}_{{dt}}^{{MC}}$. *S*_*MC*_ represents the dose deposited by primary radiation in the centre voxel, *ρ* is the density of the object, and $\hat{{\mathrm{RSP}}}$ is the reconstructed stopping power.

### 2.3. Spatial resolution: pixel size and scattering effect

In proton imaging, the spatial resolution limitation is mostly dictated by the width of the scattering distribution, often imposing a stricter limit on the modulation transfer function than the detector pixel size and pixel sampling frequency. However, for heavier ions, this limitation is less important. The spatial resolution degradation due to the scattering is calculated as the Fourier transform of the Gaussian scattering distribution, and the metric used to measure it is often the 10% level (Rit *et al* 2013). Concretely, the Gaussian spread of the MTF in the frequency domain can be expressed, accounting for sampling, scattering, and reconstructed pixel size, as:

\begin{eqnarray*}\mathrm{MTF}(\epsilon )=\mathrm{sinc}(\epsilon a)\exp (-2{\pi }^{2}{\sigma }_{{\mathrm{scatt}}}{\left({\bf{r}}\right)}^{2}{\epsilon }^{2}) \circledast {\mathrm{III}}_{1/a},\end{eqnarray*}

where *a* represents the pixel size, *σ*_scatt_ is the scattering distribution defined in equation (4), and III_1/*a*_ is the sampling frequency (comb function) for a uniform array of pixels. The latter imposes a strict threshold to the reconstructed spatial resolution at the Nyquist frequency, which is calculated as *f* = 1/2*a*. For comparison purposes, we will focus on both the intrinsic resolution from the scattering spread, and the complete description of the spatial resolution including the comb (which represent the sampling frequency) and sinc functions (which represent the pixel size impact). To show clearly the impact of scattering distribution and limitation by the comb/sampling, the pixel size is fixed at 0.25 mm in figures related to spatial resolution only for demonstration purposes. For SNR and dose metrics, the pixel size is fixed at 1 mm as described above.

### 2.4. Geant4 MC simulations

Monte Carlo simulations were carried out to produce projection data to validate the model described in this paper. MC simulations in this work were implemented using Geant4 MC code version 10.1.1 (Agostinelli *et al* 2003).

#### 2.4.1. Physics package

In this work, nuclear elastic and inelastic interactions are considered exclusively for the dose they deliver and the fluence they remove from the beam, but are tagged and removed when evaluating the noise and image quality. The model aims to represent electromagnetic interactions only and the introduction of nuclear interactions would introduce unnecessary uncertainties against the goal of the model. Furthermore, it is expected that nuclear interactions can be filtered out of the signal using the recent *dE*−*E* filter developments proposed by Volz *et al* (2018) for hadron imaging, and would affect only the noise. The processes considered include electromagnetic energy loss and straggling (following Bethe-Bloch theory) and MCS based on Lewis theory (Goudsmit and Saunderson 1940) using the Urban model (Urban 2006) as well as elastic/inelastic nuclear interactions. In precise terms, for all particles the following physics lists were used: (1) the standard electromagnetic option 3 for high accuracy of electron and ion tracking and (2) the ions elastic model (G4HadronElasticPhysics). For inelastic interactions: in protons the light binary cascade model (G4IonBinaryCascadePhysics) was used with standard Tripathi cross-section (Hall *et al* 2016), in helium, the same model was used but with the modified Tripathi cross-section from Horst *et al* (2017), and the quantum-molecular-dynamics (QMD) was the model used for all heavier ions (G4IonQMDPhysics) with Shen cross-section (Dudouet *et al* 2014), following the most up-to-date recommendations. The decays module was used for all ions (G4DecayPhysics). Step limiter cuts were set to 1 mm.

#### 2.4.2. Beam setup

For each ion beam species studied, *n* = 10^7^ particles were simulated. Two methods of determining beam input energy were used. First, an energy necessary to produce a range of *R* = 26 cm in water was used (see table 1 for detailed energy values). Second, a clinically relevant spatial resolution was fixed (10 lp cm^−1^) and the energy was chosen following two conditions in order of importance:

(i) Particle energy must allow crossing of a 20 cm radius cylinder while keeping an exit energy >70 [MeV/*u*] (Arbor *et al* 2015) to yield energy independent RSP.
(ii) Particle energy must render a MTF_10%_ as close as possible to 10 lp cm^−1^ while respecting condition 1.

Details of the required energy values to fulfill these conditions are given in table 2. The initial beam flux was distributed evenly along the lateral side of the simulation world, centred on the water cylinder. No initial angular deviation was given to the particles.

**Table 1.** Monte Carlo Parameters to calculate the dose as a function of the SNR ratio for various ions calculated for a range of *R* = 26 when crossing a cylinder of 20 cm. The range was chosen to mimic current detector developments (Bashkirov *et al* 2009).

		Proton	Helium	Lithium	Boron	Carbon
		${}_{1}^{1}{\mathrm{H}}$	^4^_2_He	^6^_3_Li	${}_{5}^{10}{\mathrm{B}}$	${}_{6}^{12}$Ca
*E*_*init*_	(MeV/*u*)	200.0	200.0	253.9	345.7	386.9
*σ*_*WET*_	[mm]	2.52 ± 0.10	1.26 ± 0.06	1.01 ± 0.05	0.75 ± 0.04	0.66 ± 0.04
${g}_{{ph}}^{{MC}}({\sigma }_{{nuc}})$	(—)	1.12 ± 0.10	1.28 ± 0.17	1.40 ± 0.20	1.53 ± 0.23	1.59 ± 0.23
${g}_{{dt}}^{{MC}}({\sigma }_{{nuc}})$	(—)	1.08 ± 0.07	1.20 ± 0.14	1.27 ± 0.17	1.32 ± 0.18	1.34 ± 0.19
*S*_*MC*_	[MeV mm^−3^]	1.13 ± 0.05	4.86 ± 0.21	8.33 ± 0.36	11.16 ± 0.52	13.18 ± 0.55
*E*_*out*_	[MeV/*u*]	87.0	87.0	108.9	145.5	161.5

**Table 2.** Monte Carlo Parameters to calculate the dose as a function of the SNR ratio for various ions calculated for an MTF_10%_ of 10 lp cm^−1^, with the minimum energy to cross a 20 cm diameter cylinder while keeping an exit energy >70 MeV/*u*.

		Proton	Helium	Lithium	Boron	Carbon
		${}_{1}^{1}{\mathrm{H}}$	^4^_2_He	^6^_3_Li	${}_{5}^{10}{\mathrm{B}}$	${}_{6}^{12}$Ca
*E*_*init*_	(MeV/*u*)	350.0	191.1	234.6	309.6	343.1
Range	(cm)	66.2	24.1	22.8	21.7	21.4
MTF_10%_	(lp cm^−1^)	10.0	10.7	12.9	16.5	18.0
*σ*_*WET*_	(mm)	4.50 ± 0.17	1.20 ± 0.05	0.92 ± 0.04	0.67 ± 0.03	0.61 ± 0.03
${g}_{{ph}}^{{MC}}({\sigma }_{{nuc}})$	(—)	1.11 ± 0.09	1.28 ± 0.17	1.42 ± 0.21	1.55 ± 0.23	1.61 ± 0.24
${g}_{{dt}}^{{MC}}({\sigma }_{{nuc}})$	(—)	1.52 ± 0.23	1.14 ± 0.11	1.13 ± 0.10	1.09 ± 0.08	1.08 ± 0.07
*S*_*MC*_	(MeV mm^−3^)	0.77 ± 0.03	5.5 ± 0.23	9.75 ± 0.41	13.82 ± 0.57	15.47 ± 0.61
*E*_out_	(MeV/*u*)	281.4	70.0	70.3	71.4	70.9

#### 2.4.3. Simulation world and detector construction

The simulation world was defined as a 30 × 30 × 30 cm^3^ air box in which the 20 cm diameter water cylinder sits in the middle, with height matching the world’s height. A pixel size of 1 mm was chosen to acquire radiographs for noise measurements. Particles were recorded at the plane of intersection crossing the middle voxel of the phantom, to measure the noise in the middle of the phantom.

A water tank was used to mimic an energy loss detector, such as a calorimeter or a range telescope. The water tank was placed at the distal edge of the phantom, with width and height matching that of the simulation world (30 cm). Its depth was adjusted to be greater than the particle range in every simulation scenario (see tables 1 and 2).

#### 2.4.4. Parameters acquisition

For each ion species, the various parameters required to calculate the SNR/dose relationship (equation (10)) were acquired as follow: *E*_*init*_ was calculated in two ways: (1) from the Geant4 definition of stopping power to obtain a fixed range of *R* = 26 cm in water for each ion species, following equation (1), or (2) to fulfill the conditions in section 2.4.2. *σ* *_WET_* was measured from radiographs reconstructed at a tracker plane placed in the middle of the phantom using only primary electromagnetic interactions. The nuclear attenuation in the phantom, *g*_*ph*_^*MC*^, was calculated by taking the ratio of the number of primary particles in the middle of the water cylinder to the number of primaries at the distal detector. The nuclear attenuation in the detector, *g*_*dt*_^*MC*^, was calculated by first simulating a beam of particles (with *E* = *E*_out_, see table 1) impinging on a water cube, and then by calculating the ratio of primary particles at the end of the range to the initial number of particles. Finally, S_*MC*_ was calculated by taking the ratio between the total energy deposited in a 1 mm^3^ cube located in the centre of the phantom and the number of primaries crossing that location.

#### 2.4.5. Error analysis

In this work, error analysis was calculated on discrete variables (*g*_*ph*_^*MC*^ and *g*_*dt*_^*MC*^) and on continuous variables (*S*_*MC*_ and *σ*_*WET*_). For the discrete variables, the binomial variance was used ($\sigma ={p}_{{\mathrm{eff}}}^{* }(1-{p}_{{\mathrm{eff}}}))$ where *p*_eff_ represents the fractional attenuation and is represented as *p*_eff_ = ∫*σ*_*nuc*._(*E*)*p*(*E*)*dE*, where *σ*_*nuc*._(*E*) is the nuclear probability density as a function of energy and *p*(*E*) represents the energy distribution of the particle beam at this depth. For the continuous variable (*S*_*MC*_ and *σ*_*WET*_), the standard deviation was found from the square of the expectation of the values, and the expectation of the square (i.e. $\sigma (X)=\sqrt{E[{X}^{2}]-(E{\left[X\right]}^{2}}$). The uncertainties were propagated assuming uncorrelated errors, i.e. $\sigma ({D}_{c})=\sqrt{{\sum }_{i}{\left(\partial {D}_{c}/\partial {A}_{i}\right)}^{2}{\sigma }_{{A}_{i}}^{2}}$ where *A*_*i*_ represent the various variables in equation (10). Due to the inherent uncertainties within the cross-section models, we elected to display only two significant figures of precision in our average results.

## 3. Results

**Figure 2.**
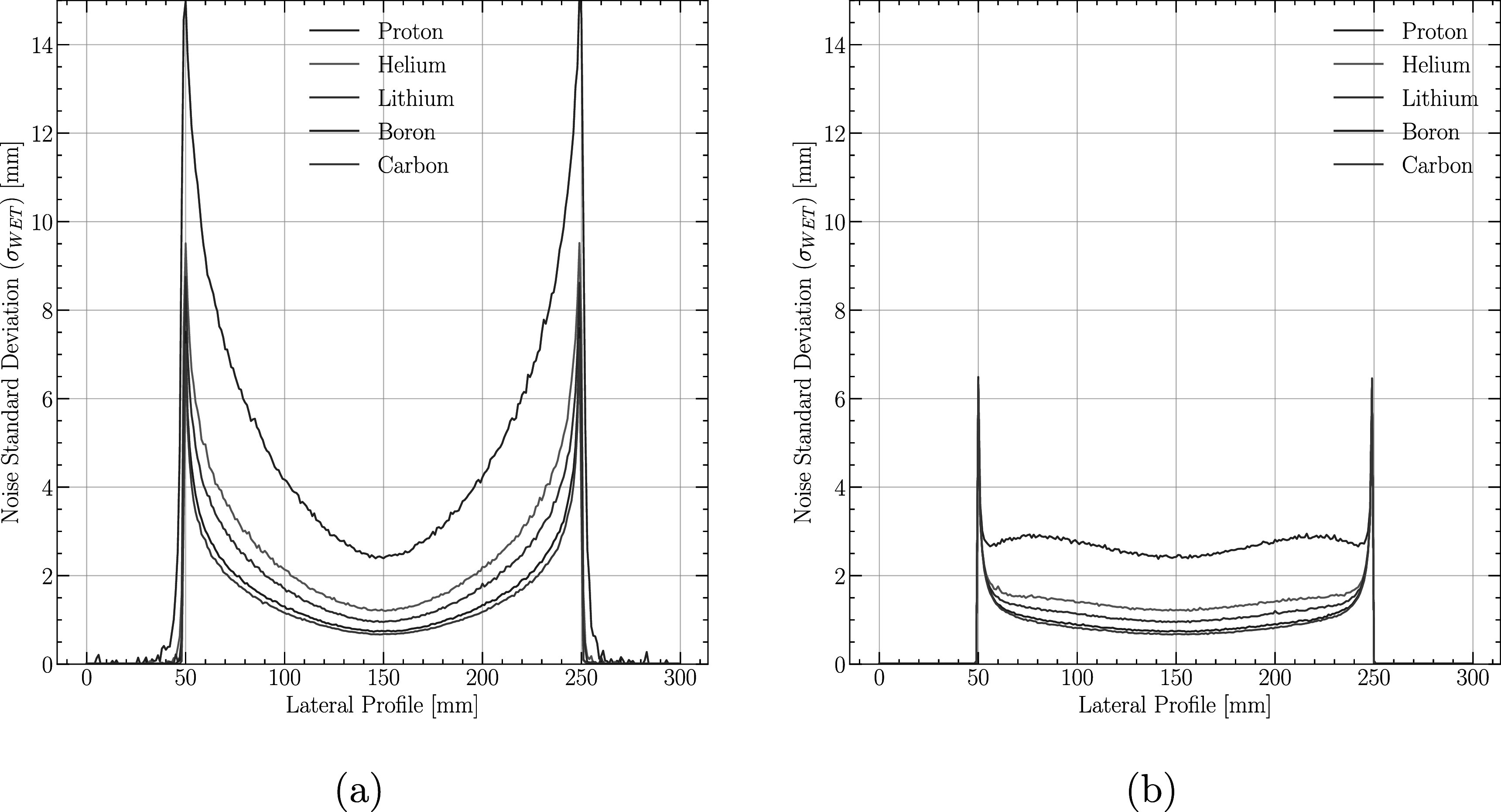
(a) Electromagnetic noise (MCS and straggling) for all ions investigated in this study recorded at the rear tracker for a 26 cm thick water cylinder. (b) Electromagnetic noise (MCS and straggling) for all ions investigated in this study recorded at the front tracker. Of note is the lateral bump in the proton noise (b), which comes from increased scattering around the edges. The sharp peak at the edges of the cylinder originates from the overlap of the cylinder producing a combination of ions having crossed air and object.

In figures 2(a) and (b), we demonstrate the electromagnetic noise at respectively the rear and the front tracker (to show noise behaviour throughout the phantom) for all chosen ion species for a fixed range of *R* = 26 cm for particles crossing a water cylinder of 20 cm diameter. The noise in proton imaging is, as expected, much larger in the rear tracker than in the front tracker due to the scattering effects detailed in Collins-Fekete *et al* (2020). This feature holds true for heavier ions but is of lesser importance due to the decreased scattering. Of note, one sees two bumps located symmetrically around the middle of the noise profile for front tracker in proton radiography (figure 2(b)). This feature has been examined in our previous publications, as well as in Rädler *et al* (2018), and is caused by the scattering noise, which increases towards the edge of the object. It is also of interest to note that this behaviour disappears for heavier ions due to the reduction of the scattering distribution for such particles. Both sharp lines around the edges of the cylinder (at 50 and 250 mm in the lateral profile) come from pixels in the detector, for which the projection sharp edge of the cylinder overlap producing a combination of object and air.

Each of the individual parameters needed to calculate equation (10) for a fixed range have been calculated through Monte Carlo simulation, as detailed in section 2.4, and are presented in table 1.

**Figure 3.**
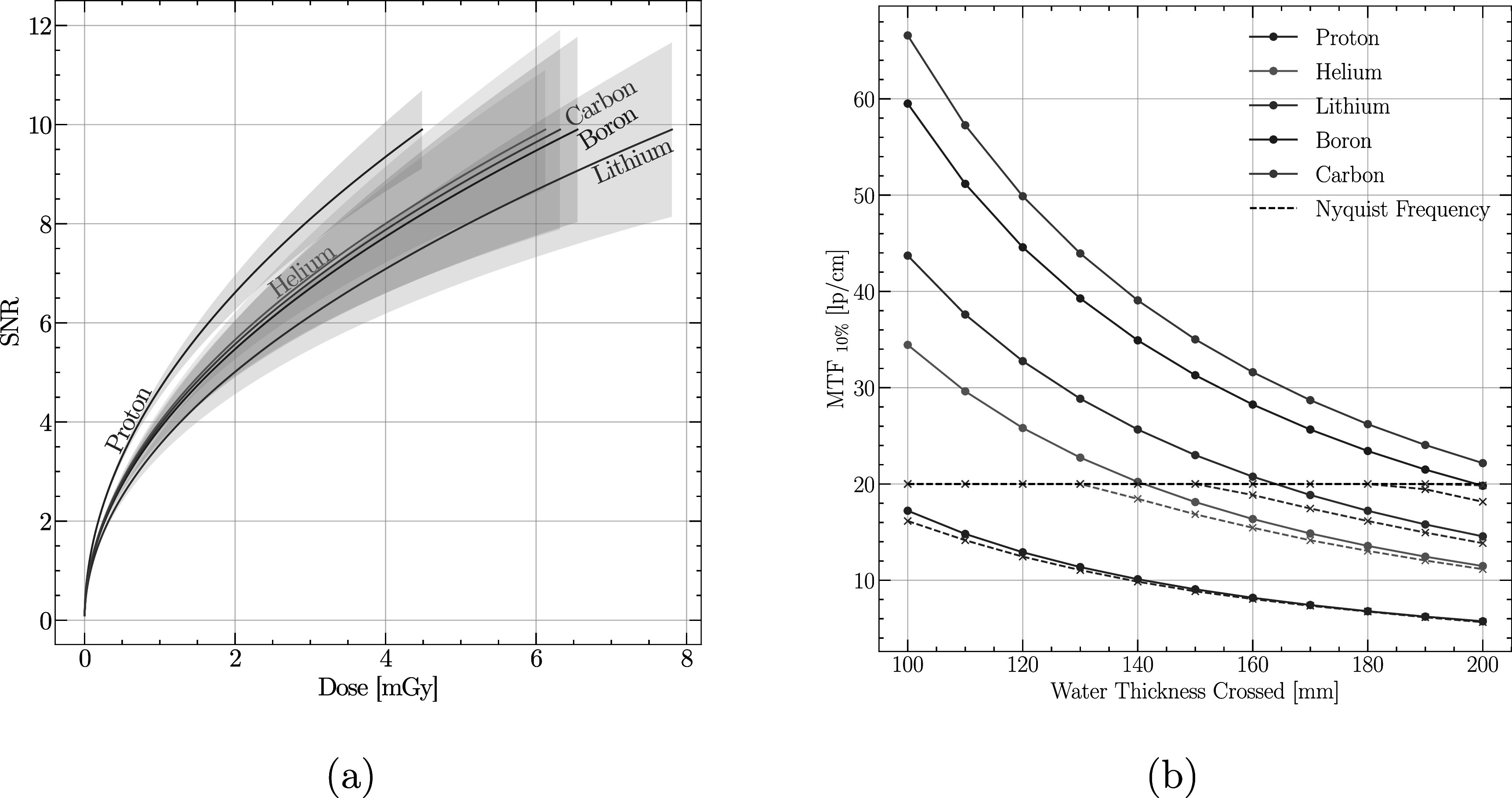
Image quality metrics of interest for energy-loss tomography for various ions. (a) SNR against dose for various available ions species (equation (10)). The signal to noise ratio is acquired at a fixed range of *R* = 26 cm for particles crossing a 20 cm WET cylindrical water object. The shaded area represents 1 standard deviation around the calculated values. Pixel size is fixed to 1 mm. (b) Scattering-only (full line) and complete (dotted line) MTF_10%_ for a set of ion species available for imaging crossing water slab of thickness ranging from 10 to 20 cm. Pixel size was fixed to 0.25 mm. Initial energy is set to ensure a fixed range of *R* = 26 cm (table 1).

Figures 3(a) and (b) demonstrate respectively the SNR to dose relationship, and the modulation transfer function for a tomograph produced with the various ions investigated here. Although shown separately, these two quantities are correlated through the minimum usable pixel size which is often dictated by the scattering distribution. It can be seen that the lowest nuclear charge produces the highest SNR (proton) for a given dose, but that this also leads to the lowest spatial resolution.

For a fixed range, which correlates to a fixed noise (*σ*_*WET*_), the *D*_*c*_ to SNR ratio is constant between ions. Thus, for a range of *R* = 26 cm, helium requires 36% more dose to reach the same SNR, 75% for lithium, 46% for boron and 40% for carbon. An interesting result of figure 3(a) is the fact that carbon and boron particles have a higher SNR than lithium. We hypothesize that this is caused by the interplay between carbon’s higher nuclear attenuation in the phantom/detector and its minimal noise level when compared to other ions. lithium has, therefore, the worst of both worlds, with both high noise and high nuclear attenuation, making it the ion that provides the worst SNR in our model.

Furthermore, figure 3(b) demonstrates the spatial resolution limitation of particle imaging induced by Coulomb scattering, either individually (full line) or with pixel sampling and size effects (dotted line). The calculated spatial resolution for proton imaging is 5.73 (lp cm^−1^) for 200 MeV protons crossing 20 cm of water, in-line with what has been found in the literature (Li *et al* 2006). In comparison, conventional x-ray CT systems usually limit their spatial resolution to 10–11 (lp cm^−1^) for noise considerations, a level that is reached by helium imaging for 200MeV particles crossing 20 cm of water. However, when looking at heavier ions or smaller thickness crossed, we expect pixel size and pixel sampling to start to become important in comparison with scattering. This is observed in the dotted line of figure 3(b) where both the Nyquist limit of the comb function and the attenuation of the sinc function affect the spatial resolution, effectively limiting it at $f=\tfrac{1}{2a}$. When considering scattering only and for a fixed range, the MTF_10%_ of helium imaging is roughly 2 times higher than that of proton, 2.5 times for lithium, 3.5 times for boron and 3.9 times for carbon.

These results seem to indicate that protons is an optimal choice for SNR, and carbon for spatial resolution. However, it is an unfair comparison, as the proton’s 5.0 lp cm^−1^ might not be acceptable clinically, no matter the SNR. It is of interest to look at a scenario in which we fixed the spatial resolution to be clinically relevant (i.e. equivalent to that of x-ray CT) and compare the SNR for each investigated ion. Thus, we fixed it to 10 lp cm^−1^ following the conditions defined in section 2.4.2 and calculated other relevant parameters (table 2).

**Figure 4.**
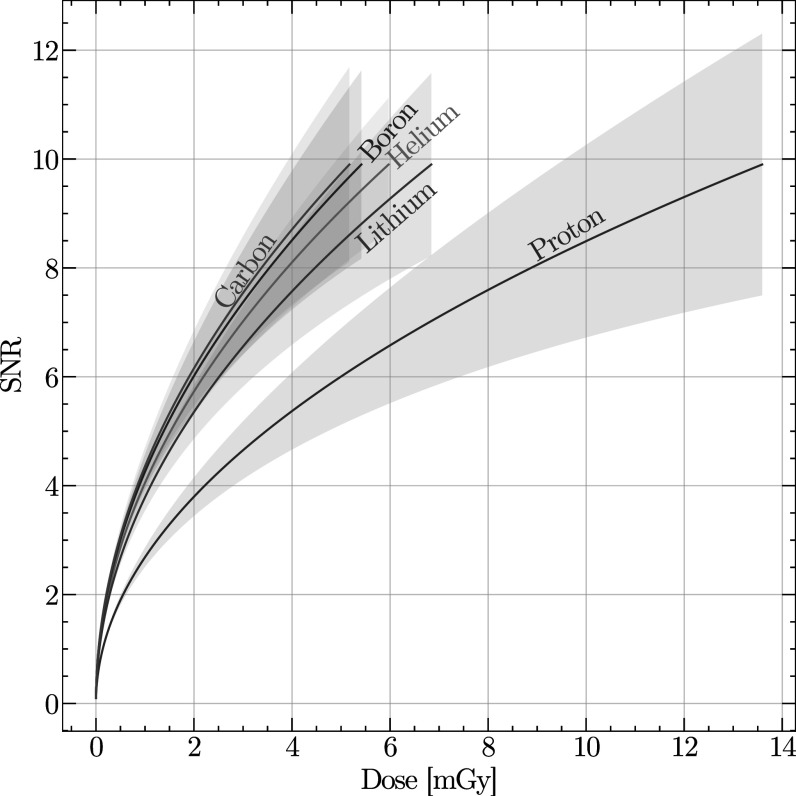
SNR against dose for various available ions species (equation (10)) for energy-loss tomography for various ions following fixed conditions for a 10 lp cm^−1^ spatial resolution (see section 2.4.2) with a 1 mm pixel size. The shaded area represents 1 standard deviation uncertainty around the calculated values. Parameters to generate this graph are outlined in table 2.

Results from equation (10) for parameters detailed in table 2 are shown in figure 4 where it can be seen that at a fixed 10 lp cm^−1^, protons perform particularly poorly. This is caused by (1) the increased noise originating from the elevated entrance energy, and (2) the increased loss of primaries in the detection due to the longer range required to stop the particles in a detector.

## 4. Discussion

This manuscript builds on our previous work that developed a framework for describing the statistical effects occurring when passing a beam of protons through a uniform object and their effects on the fundamental image quality metrics for proton imaging. This manuscript extends this work to ions commonly considered for ion tomography. The work follows the themes of spatial resolution and dose against SNR, which are intricately related as shown here and in our previous study (Collins-Fekete *et al* 2020). In this study, the signal coming from electromagnetic energy loss, and the related noise, are considered for image formation, whereas secondary particles are considered for their impact on the extra dose delivered to form an image and the consequent fluence lost in creating them.

In charged particle imaging, spatial resolution is often degraded by the scattering of the particles due to Coulomb scattering throughout their trajectories. This scattering is mostly dictated by the velocity and the charge over mass ratio (Collins-Fekete *et al* 2020). For these reasons, for a fixed range, heavier ions scatter less than protons, which translates to a higher spatial resolution when ignoring detector pixel size and sampling frequency effects (Plautz *et al* 2016). This is demonstrated in figure 3(b) when considering only the scattering effects (full line). However, as heavier particles tend to exhibit a sharper point-spread-function, this increased spatial resolution becomes rapidly limited by the image receptor pixel size. In our previous manuscript, we discussed the limitation on pixel size imposed by the scattering distribution. This can be seen directly in figure 3(b), where the Nyquist frequency for 0.25 mm pixel size is above the scattering distribution limit. The 0.25 mm pixel size is chosen to represent what is typically achieved in the field (Bashkirov *et al* 2016). This limitation imposes a strict correlation between spatial resolution and SNR, as the pixel size also strongly influences the SNR/dose relationship.

When comparing the SNR, addressing the still open question (Gehrke *et al* 2018), this work demonstrates that, for a fixed range, the SNR of protons is higher than that of other commonly used ions for radiotherapy. Common perception would suggest that the noise in proton CT from straggling and scattering balances out the additional dose in helium CT, however, this interpretation neglects the fluence loss both in the phantom and in the detector, as well as the extra dose from secondary fragments that occurs when using heavier ions. One can see (table 1) that in a 20 m water phantom, helium ions suffer a fluence loss in the phantom (*g*_*MC*_^*ph*^) 15% higher than that of protons, and receive an additional 7.5% dose above the expected electromagnetic scaling, due to the extra dose from the secondary nuclear interactions. Both these factors decrease the SNR for a fixed dose. On the other hand, if one requires a more clinically acceptable spatial resolution (10 lp cm^−1^), the scattering limitations of proton imaging quickly overcomes its benefits and protons provide the lowest SNR (figure 4).

The relationship portrayed in this manuscript are, however, in-line with what was reported by Meyer *et al* (2019). Specifically, they observe that proton CT might have lower RSP errors in uniform soft-tissue compared to helium and carbon ions, whereas it performs worst in heterogeneous tissues. These results originates from the larger scattering distribution of protons, which leads to an increased scattering noise in non-uniform objects. This conclusion is in-line with both what is demonstrated in figures 2(a)–(b) and the severe decrease of SNR experienced by the protons when imposing a clinically relevant spatial resolution (figure 4).

**Figure 5.**
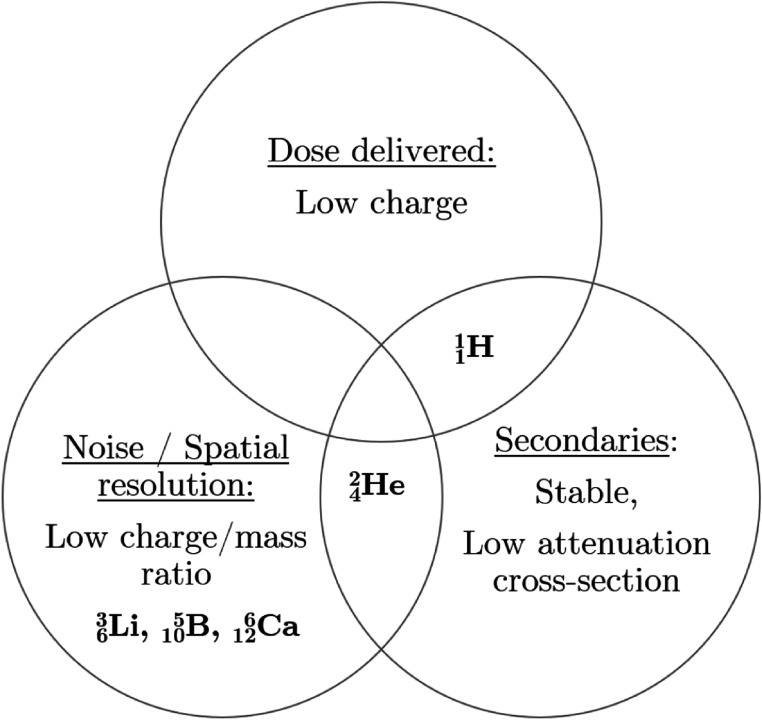
This Venn diagram summarizes the three important characteristics to maximize image quality metrics in particle tomography. Ions species considered in this study are shown in the diagram. Few ions species fulfill two of these criteria, e.g. protons have both a low electromagnetic dose and a low nuclear attenuation cross-section but have a large scattering distribution. In conclusion, no particle can fulfill all three characteristics combined.

Using the results observed in this manuscript, we can explore the properties of different ions and how these affect different image quality metrics. To optimize the spatial resolution and noise, the ion should have the lowest nuclear charge to mass ratio, and the highest energy per mass unit. Therefore, heavy ions are generally favoured for spatial resolution. To optimize the SNR against dose, the ion should deliver the lowest dose per particle, and thus have the lowest nuclear charge possible and the highest nuclear stability. In this case, light ions are favoured. Finally, the ion should be stable enough and have a low cross-section for nuclear decay to maximize the number of primaries by the detector at the distal end. These considerations are summarized in figure 5. This is a classic concept of ‘No such thing as a free lunch’, as no single ion fulfils all these considerations and a compromise must be made. Whereas proton imaging provide the highest SNR, helium and heavier ions provides higher spatial resolution.

It is important to mention here that we did not include direct measurement of noise in the detector. It should not change the results in the fixed range comparison for the following reasons: this study investigates the comparison of SNR between different ion species, and the noise in the detector would be scaled by this additional factor, but the relative numbers would remain similar (see Gehrkre *et al* (2018) for detailed considerations). Furthermore, the noise properties of a detector are highly dependent on its construction design, and we wished to remain clear of any biasing choice. In addition, we assumed every product from the nuclear reactions can be perfectly filtered out. This assumption was made to simplify the comparison between ions. In practice, this process will not be not perfect, e.g. energy-loss due to charge-preserving nuclear reaction would not be well rejected by this filter. We have decided not to model a filter since (1) current filters reject a large majority of these reactions and (2) any increased noise due to secondary nuclear products would support the conclusions found here, i.e. stable ions with low charge produce the highest SNR for a given dose and a fixed range.

In this study, detector were considered by explicitly modelling the fluence loss in a water tank, which represents an energy-loss measurement through a slowing down process. This basis is justified by current construction of ion imaging systems, that require particles to stop within the detector for their energy to be measured. Proposed prototype systems such as time-of-flight detectors (Worstell *et al* 2019) could theoretically remove this limitation, and one could consider using higher energy to improve the spatial resolution of an image. However, as seen when comparing figure 3(a) to 4, increasing the energy leads to a drastic decrease in the SNR.

The results produced here are limited by precision of the model of cross sections for the various ions and interactions involved. As such, the results should not be taken as absolute results, but should rather serve as a relative comparison of image quality metrics between ions. We have decided to use a simple symmetrical phantom as it allows us to derive a direct relationship between SNR and dose, which represents well the conceptual problem and provides us with a mean for direct comparison between ion species. It is important to mention that the SNR defined here is valid within the centre of our uniform cylinder, which is a simplified representation of a human body. Within a non-homogeneous body, the scattering noise is expected to increase around high-gradient inhomogeneities, such as nasal cavities, with a more pronounced increase for lower charge/mass ions with a larger scattering distribution. Thus, the SNR benefits of proton ions at lower energy are expected to be reduced in the human body.

Results presented here focus on the SNR/dose relationship and spatial resolution in a tomograph. The noise characteristics of tomography and radiography differ by the multiplicative factor in equation (9) ($\sqrt{3{{Ma}}^{2}/{\pi }^{2}}$) that accounts for all angles and is similar for all ions. On the other hand, the spatial resolution in hadron tomography is a sampling of the scattering distribution at a given depth, whereas in hadron radiography, it is the collapsing of that distribution on a single plane (Volz *et al* 2020). Since none of these differences involves the ion charge, mass, or velocity, the relations drawn for tomography can be extrapolated to radiography.

Results published in our last paper (Collins-Fekete *et al* 2020) suggested that the proton energy was a defining metric for image quality, as low energy would provide high SNR and, low spatial resolution, and vice-versa for high energy. It seems that in light of ion imaging, the picture becomes more complex, with light ion at low energy being optimal for SNR and heavy ions at high energy optimal for spatial resolution. However, protons require a significant boost in energy to produce a spatial resolution equivalent to that of x-rays, reducing considerably their SNR. In contrast, ions can produce an image with an equivalent spatial resolution while providing high SNR, with the choice of imaging parameters informed by the model presented here.

## 5. Conclusion

A study has been made to compare various ions against conventionally established image quality metrics, i.e dose, signal to noise ratio and spatial resolution. It was found that protons demonstrate the highest SNR for a fixed dose due to their nuclear stability and low dose delivered per particle. On the other hand, heavier ions such as carbon yield the highest spatial resolution due to their high energy required to cross the patient and their reduced scattering due to their low charge to mass ratio. Finally, when fixing a clinical spatial resolution, proton ions seems to perform poorer than other heavier ions.

## Data availability

The datasets generated during and/or analysed during the current study are available from the corresponding author on reasonable request. Algorithms used for this study can be found at https://github.com/cacof1.
